# Assessment of Effects of Si-Ca-P Biphasic Ceramic on the Osteogenic Differentiation of a Population of Multipotent Adult Human Stem Cells

**DOI:** 10.3390/ma9120969

**Published:** 2016-11-29

**Authors:** Patricia Ros-Tárraga, Rubén Rabadan-Ros, Angel Murciano, Luis Meseguer-Olmo, Piedad N. De Aza

**Affiliations:** 1 Grupo de Investigación en Regeneración y Reparación de Tejidos, UCAM—Universidad Católica San Antonio de Murcia, Guadalupe, 30107 Murcia, Spain; p.ros.tarraga@gmail.com (P.R.-T.); rubenrabadanros@gmail.com (R.R.-R.); 2 Departamento de Materiales, Óptica y Tecnologia Electrónica, Universidad Miguel Hernández, Avda. Universidad s/n, 03202 Elche (Alicante), Spain; amurciano@umh.es; 3 Service of Orthopaedic at Arrixaca University Hospital, UCAM—Catholic University of Murcia, 30120 Murcia, Spain; lmeseguer.doc@gmail.com; 4 Instituto de Bioingenieria, Universidad Miguel Hernandez, Avda. Ferrocarril s/n. Elche, 03202 Alicante, Spain

**Keywords:** bioceramic, calcium silicophosphate, eutectoid, adult human mesenchymal stem cells, cell culture, osteogenic differentiation

## Abstract

A new type of bioceramic with osteogenic properties, suitable for hard tissue regeneration, was synthesised. The ceramic was designed and obtained in the Nurse’s A-phase-silicocarnotite subsystem. The selected composition was that corresponding to the eutectoid 28.39 wt % Nurse’s A-phase-71.61 wt % silicocarnotite invariant point. We report the effect of Nurse’s A-phase-silicocarnotite ceramic on the capacity of multipotent adult human mesenchymal stem cells (ahMSCs) cultured under experimental conditions, known to adhere, proliferate and differentiate into osteoblast lineage cells. The results at long-term culture (28 days) on the material confirmed that the undifferentiated ahMSCs cultured and in contact with the material surface adhered, spread, proliferated, and produced a mineralised extracellular matrix on the studied ceramic, and finally acquired an osteoblastic phenotype. These findings indicate that it underwent an osteoblast differentiation process. All these findings were more significant than when cells were grown on plastic, in the presence and absence of this osteogenic supplement, and were more evident when this supplement was present in the growth medium (GM). The ceramic evaluated herein was bioactive, cytocompatible and capable of promoting the proliferation and differentiation of undifferentiated ahMSCs into osteoblasts, which may be important for bone integration into the clinical setting.

## 1. Introduction

Despite its extraordinary healing ability, bone response may be unsuccessful for repairing the severe damage caused by injuries, tumours, infections and ageing-related problems. The surgical techniques applied for bone reconstruction are based on bone grafting procedures.

Currently, materials available for bone replacement include autologous bone, allograft, xenograft, demineralised bone matrix (DBM), and various synthetic materials such as ceramics Hydroxyapatite (HA), tricalcium phosphate (TCP), composites, polymers, silk-fibroin, etc., and all with the associated complications and drawbacks. Of them all, an autologous cancellous bone graft, is considered a gold standard because of its osteoconductive, osteoinductive and osteogenic properties, which are necessary for bone repair.

Current technology enables the regeneration of viable tissue or organs by using cultured cells and suitable scaffolds. We previously reported that ahMSCs derived from bone marrow can be cultured on TCP and Si-TCP ceramics [1,2]. Under osteogenic conditions, cultured ahMSCs further differentiate into cells with the osteoblastic phenotype and are able to fabricate an extracellular bone matrix (osteopontin, osteocalcin, bone sialoprotein).

Previous research suggests that silicon, as an essential element in skeletal development. Carlisle [3,4,5] first reported in the 1970s that silicon was uniquely localized in the active areas of young bone and involved in the early stage of bone calcification. Similar studies by Schwarz and Milne [6] have shown that silicon deficiency in rats resulted in skull deformation, with the cranial bones appearing flatter than normal.

As Si performs a major function in human bone, Si has been widely incorporated into Ca-P bioceramics to enhance their bioactivity [7,8,9,10]. Typically, Si-doped HA and Si-TCP ceramics have been prepared, although both pure Ca-P ceramics and Si-doped Ca-P ceramics still have shortcomings. Previous studies have shown that sintered HA does not significantly degrade, but remains a permanent fixture, susceptible to long-term failure [11,12,13]. Although α and β-TCP ceramics are degradable at a quicker degradation rate than HA, in vivo osteogenesis of sintered α and β-TCP ceramics is far from optimal. Recently, Martinez et al. found that the in vitro weight loss of α-TCP and Si-α-TCP ceramics was 25% and 10% respectively, after 4 weeks of soaking in Kokubo’s simulated body fluid [14]. Mate-Sanchez et al. found that Si-TCP grafts exhibited better dimensional stability and increased bone-to-implant contact with a reabsorption rate ~71.5% of α-TCP and ~42.2% of Si-TCP after being implanted in vivo for 60 days [15,16,17].

Accordingly, materials that contain silicon, calcium and phosphorus are excellent candidates for preparing biomaterials with improved osteogenic properties [18,19,20]. The synthesis of Nurse’s A-phase (2Ca_2_SiO_4_·Ca_3_(PO_4_)_2_) and silicocarnotite (Ca_2_SiO_4_·Ca_3_(PO_4_)_2_) as monophasic materials, was addressed by the present authors in a previous work [21,22,23,24]. On the basis of the Nurse’s A-phase-silcocarnotite in the system Ca_3_(PO_4_)_2_-Ca_2_SiO_4_, an invariant eutectoid point at 1366 °C ± 4 °C for a composition of 28.39 wt % Nurse’s A-phase-71.61 wt % silicocarnotite, was also established [25]. The microstructure of an invariant eutectoid point is always lamellar, regardless of the phase diagram studied. The other compositions from the phase diagram have a hypo or hyper eutectoid microstructure formed by grains of a single phase within a much finer eutectoid matrix and therefore is not suitable, a priori, to be colonized by cells. There is only one eutectoid composition with lamellar microstructure, so that composition has been studied in this work.

The main goal of this study was to evaluate the initial response of the influence of Si-Ca-P biphasic ceramic with a eutectoid composition to stimulate the osteogenic process of ahMSCs in the presence or absence of an osteogenic supplement in culture media with a view to using the material for bone tissue engineering.

## 2. Results

### 2.1. Biphasic Ceramic Characterisation

**Figure 1:**
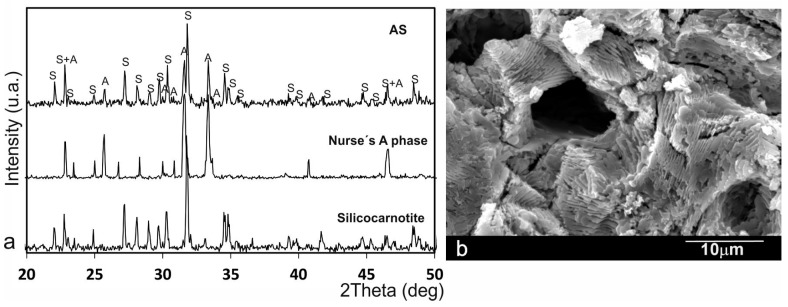
(**a**) X-ray diffraction patterns of the synthesised silicocarnotite and Nurse’s A materials and the AS eutectoid ceramic; (**b**) Scanning Electron Microscopy (SEM) image of the biphasic ceramic microstructure with a lamellae morphology.

Figure 1a shows the X-ray diffraction patterns of the synthesised powders of Nurse’s A and silicocarnotite as well as the composition 28.39 wt % of Nurse’s A-71.61 wt % silicocarnotite, referred to as AS from this point onwards. The sharp peaks and low backgrounds suggested that powders were highly crystalline. The obtained Nurse’s A-phase gave diffraction peaks, which can be assigned to the characteristic reflections of 2Ca_2_SiO_4_·Ca_3_(PO_4_)_2_ (JCPDS card No. 011-0676). Silicocarnotite could be assigned to the characteristic reflections of Ca_2_SiO_4_·Ca_3_(PO_4_)_2_, as described by Dickens and Brown [26]. The crystalline phase detected in the sample was a well-crystallised silicocarnotite (JCPDS card No. 40-393). The eutectoid AS ceramic presented a mixture of Nurse’s A and silicocarnotite phases. As the phases were solid-solution, the diffraction peaks were slightly displaced compared to the corresponding JCPDS card. Figure 1b shows a porous and homogeneous microstructure formed by very thin eutectoid platelets made up of Nurse’s A and silicocarnotite phases.

### 2.2. Monitoring ahMSCs in Culture

**Figure 2:**
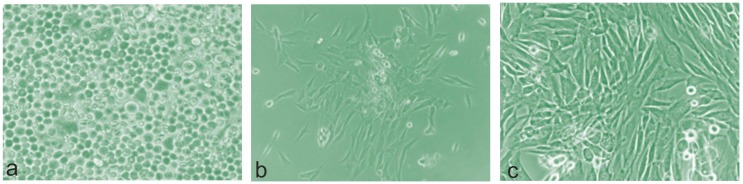
Representative phase contrast micrographs of the evolution of cultured ahMSCs. (**a**) At 3 h; (**b**) 7 days and (**c**) 28 days, adherent ahMSCs practically all displayed a fibroblastic or cuboidal morphology, depending on their confluence (magnification: ×20, ×100 and ×200 respectively).

After 3 h of culture in GM, adherent ahMSCs were observed as individual cells larger than non-adherent red blood cells or formed aggregates or colonies, and adopting a rounded or slightly elongated shape (Figure 2a). Residual was unattached around haemopoietic or dead cells that floated in the culture medium on day 7. The cells adopted an elongated and polygonal shape and formed colonies. The number of cells with cytoplasmic vacuoles was not significant, so no cytotoxic effects were observed in morphological terms (Figure 2b).

By 21 days, the culture was more confluent on the cells grown in Osteogenic medium (OM), with a similar morphology to the previous period. On day 28, practically all the adherent ahMSCs displayed a fibroblastic or cuboidal morphology, depending on their confluence (Figure 2c). No significant differences were observed when comparing both culture media. Some birefringent nodules were observed in the cultures with OM.

### 2.3. Adhesion and Morphology of the ahMSCs Seeded on AS Ceramic Discs

**Figure 3:**
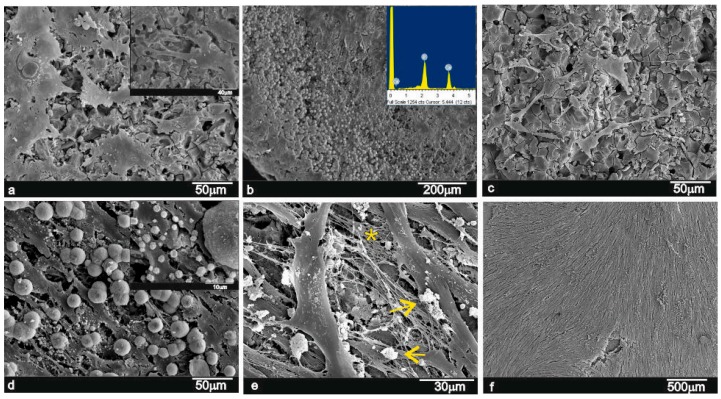
SEM images of the ahMSCs stem cells grown on ceramic surfaces after (**a**,**b**) 24 h; (**c**,**d**) 7 days; (**e**) 21 days and (**f**) 28 days in GM.

In order to show cell adhesion and morphology on the AS euctectoid ceramic, the ceramic discs cultured with ahMSCs were examined by SEM. Figure 3 representatively shows the most relevant changes observed as a result of the AS-ahMSCs interaction in culture for 24 h, and 7, 14, 21 and 28 days in GM, and for 21 and 28 days in OM.

During the first 24 h of culture in GM, cells adhered either individually or in small groups across the whole granular surface of the material (Figure 3a). The ceramic exhibited a granular layer that covered some parts of the surface, which is not an obstacle for cell adhesion or proliferation. This layer was composed of a uniform ~1-µm size, and exhibited globular structures with spicules and small nodules, both of which were composed of Ca-P elements according to the Energy Dispersive X-ray elemental microanalysis (Figure 3b and detail).

Later (7 days), the ahMSCs grown on the ceramic surfaces were seen to be polygonal in shape, although some rounded globular cells were still seen (Figure 3c). The cell number grew and was recorded as an increase in cytoplasm prolongations and cell-to-cell interactions. At this time point, the presence of mineralisation nodules suggested cell deposition continuity on AS ceramic surfaces. Granules were seen also above cells, which came into contact with their cellular membrane. This scenario suggests that granules formed simultaneously, and cells proliferated and covered the material surface (Figure 3d and inset). After the 14-day exposure period, the ahMSCs grown on the ceramic surfaces showed the characteristic flat polygonal shape, and no more relevant changes took place compared to the 7-day cultures (figure not shown). After the 21-day exposure period, fast progressive cell growth was observed on surfaces (Figure 3e). Cells formed a monolayer that partially covered the material surfaces (Figure 3e, arrows), and also produced a dense extracellular matrix by way of a fibrillar network that occupied intercellular gaps (Figure 3e, *). After the 28-day period (Figure 3f), the ahMSCs cells began to arrange in a continuous monolayer. The cell monolayer appeared to be almost continuous with the cells that presented a flattened spread morphology, in which it was impossible to recognise single cells, and covered the entire material surface.

Some mineralised nodules were also found on cell surfaces when the culture conditions were in OM. The behaviour of cells at 21 days and 28 days was strikingly similar to that in GM (figures not shown). The material showed good cell development and proliferation.

### 2.4. Cell Proliferation

The methyl tetrazolium reduction (MTT) cell proliferation assay confirmed the SEM observations, and revealed faster ahMSCs cell proliferation on the AS material from day 14 of the culture study than for the control, which progressed linearly with time from the start of the assay (*p* < 0.05).

**Figure 4:**
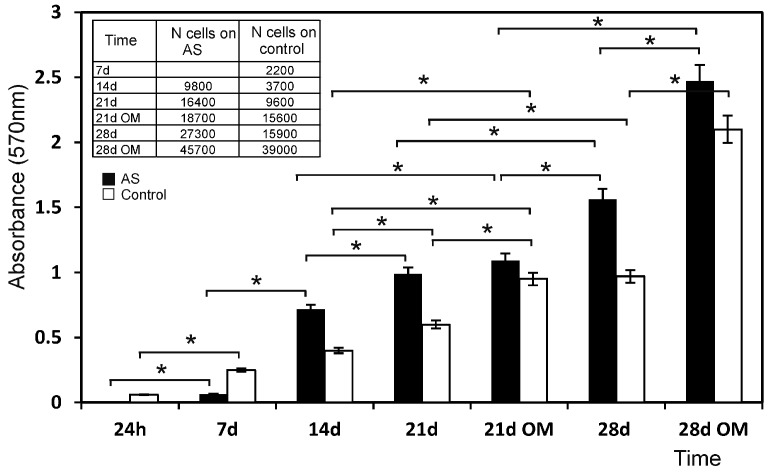
Proliferation of the cells seeded on the AS ceramic compared to plastic, used as the control in the MTT assay. * denotes significance at *p* < 0.05 between these samples.

Figure 4 shows the cell proliferation on the surfaces of specimens during the culture time in GM and OM. At 24 h, the low absorbance values indicated that a small number of cells initially adhered to the AH ceramic. After 14 days of culture, their proliferation rate overtook that of the control. From day 14 until the end of the assay, the number of cells that grew on the AS surface was always larger compared to the control. The cell population used as the control progressed linearly with time from the time the assay began. However, the proliferation rate was quite modest, and cell numbers did not duplicate.

Regarding the ahMSCs cultured in OM, and unlike the above-described results, the absorbance values of the cells cultured in OM increased with time, which indicated high cell proliferation in this medium with the assayed ceramics.

### 2.5. Alkaline Phosphatase

**Figure 5:**
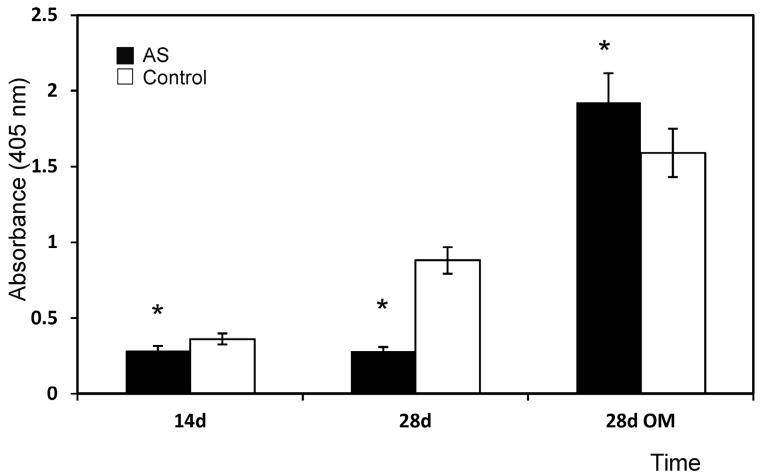
Alkaline Phosphatase quantification of the ahMSCs incubated with the AS biomaterial and for the control (plastic culture) was measured by the p-nitrophenyl phosphate (pNPP) assay after 14 and 28 days in GM and after 28 days in OM. * on the column denotes significance *p* < 0.05, for the ANOVA test.

Cell differentiation was assessed in terms of the alkaline phosphatase (ALP) activities of ahMSCs on days 14 and 28 of culture in GM and after 28 days of being cultured in OM (Figure 5). At day 14, ALP was expressed at lower levels. No significant differences were detected between the material and the control. After 28 days in GM, the enzyme activity in the control sample was significantly higher than in the AS ceramic. Substitution of GM by OM changed the results. The enzyme activity in the samples incubated with the AS ceramic was greater than that of the cells in the control, and a statistically significant difference was found on day 28.

### 2.6. Alizarin Red Staining and Activity Analysis

**Figure 6:**
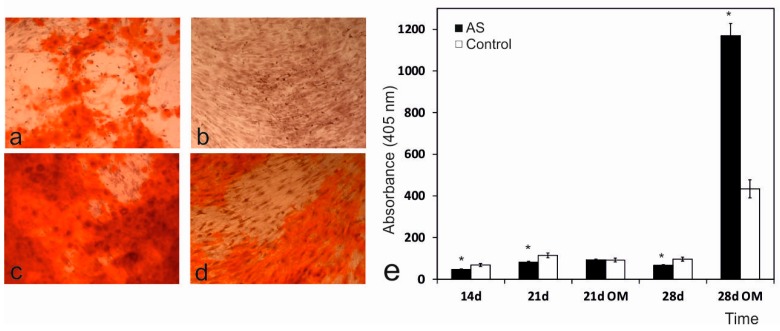
Alizarin Red staining and the activity analysis of the ahMSCs seeded on plastic and the AS ceramic on day 28. The Alizarin Red expression of the ahMSCs seeded on plastic (**a**,**b**) and AS ceramic (**c**,**d**) in either OM (**a**,**c**) or GM (**b**,**d**) (×100). (**e**) The quantitative ALP activity of the ahMSCs seeded on plastic and the AS ceramic was measured on days 14, 21 and 28 in GM and on days 21 and 28 in OM. * indicates significant differences, *p* < 0.05.

The Alizarin Red expression of the ahMSCs cultured on plastic and AS eutectoid discs in both the growth and osteogenic media was examined. As shown in Figure 6a–d, Alizarin Red staining was more intense for the ahMSCs on the AS eutectoid discs than those on plastic in both GM and OM on day 28. Modest red staining was identified in GM, which suggests that calcium crystal deposition in the ceramic had commenced. The control monolayers incubated with GM showed no staining (Figure 6b). A strong red stain appeared in OM (Figure 6c), compared with GM (Figure 6d), when ahMSCs were cultured on the AS eutectoid discs. Similarly, the quantitative examination data analysis showed that the Alizarin Red activity of the cells cultured on the AS ceramic discs increased with time throughout the assay period, with a higher value in OM and a statistically significant difference on day 28 (Figure 6d).

### 2.7. Surface Markers in the Cells Seeded on the AS Ceramic 

**Figure 7:**
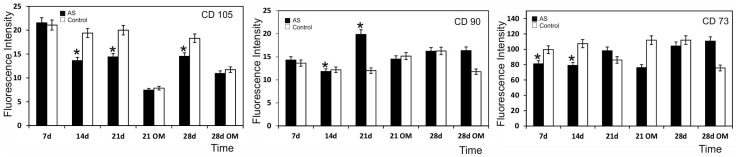
CD105, CD90 and CD73 expressions of the cells seeded on the biomaterial compared to the control. Data represent the fluorescence level. The experiment was performed in triplicate. * denotes a significant difference at *p* < 0.05 between the cells cultivated on the material and the control at the same time.

The ahMSCs of both cultures in the presence of the material and control were negative for all the analysed haematopoietic markers (data not shown). The cluster differentiation (CD) markers, CD105, CD90 and CD73 expressions of the cells seeded on the biomaterial compared to the control are shown in Figure 7. Starting from day 14, the CD105 expression of the cells seeded on the AS ceramic and cultured in GM was always significantly lower than the control, but was comparable during the first 7 days in culture; the CD105 expression lowered with time and was significant at all the time points. When OM was added, the antigen expression became significantly lower than the control. These data demonstrate that the activation of a differentiation programme was accompanied by a lowered expression of the 105 marker characteristic of MSCs.

During the 28-day study, the CD105 marker expression in GM did not significantly change compared to the 21-day period in culture. Possibly, cells retained a minimal level of expression of this marker. The differences between the 21-day period in OM and the 28-day one in OM were not statistically significant. We conclude that CD105 expression did not change after day 21 (Figure 7).

CD90 expression did not change significantly with either time or addition of OM, although we highlight that its expression was reduced for the material and control samples after 21 days in OM, which was the time point when cells entered the mature phase of the differentiation process (Figure 7). CD73 expression did not significantly change from day 1 to the last day of the study (Figure 7). By 21 days, significant differences were observed between the control and the ceramic material, the control values remained the same, and the values of the samples incubated with the biomaterial increased.

### 2.8. The Ions Released from the AS Ceramic and Their Effect on ahMSCs

**Figure 8:**
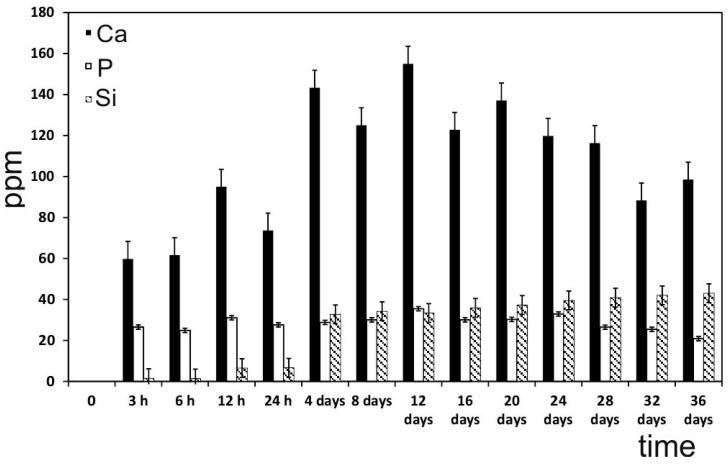
The Si^4+^, Ca^++^, HPO_4_^=^ concentrations in the ahMSC culture medium after different immersion times.

The release of soluble Si, Ca and P elements by dissolution of the ceramics in relation to the immersion time is shown in Figure 8. Initially, the calcium (Ca) concentration significantly increased during the first 12 h of exposure and reached the maximum concentration after 12 days of immersion. After day 12, the Ca concentration dropped to 155 ppm, but the final concentration was still 63.54% higher than the original one. The silicon (Si) concentration increased from the first 3 h and continued to grow until the end of the experiment, which indicated that the AS ceramic was dissolved in the medium. The phosphorus concentration (P) changed slightly for the whole immersion time, which suggests that the consumption rate of P ions due to the Ca-P layer precipitation was similar to the P ion release rate of the ceramics.

## 3. Discussion

For the bone regeneration and bone tissue engineering applications, an ideal biomaterial scaffold designed for bone tissue engineering requires osteoconductive, osteoinductive and osteogenic properties. An optimal biomaterial used as a bone substitute should not only be a temporary scaffold used to support the adhesion, growth, proliferation and differentiation of “seed” cells (such as ahMSCs), but should also be able to degrade over time into non-toxic products, which can be metabolised by physiological mechanisms.

Adding inorganic elements to scaffolds could significantly improve the bioactivity of materials, as previously confirmed in bioglass, glass-ceramic, and bioceramics that contain CaO, SiO_2_ and P_2_O_5_ [27,28]. Given the effect of these silicate-based materials on promoting cell proliferation and osteogenic differentiation, we synthesised in this study a biphasic Si-Ca-P ceramic for the first time. The ceramic obtained herein had a homogeneous porous microstructure with a lamellae morphology of Nurse’s A and silicocarnotite phases (Figure 1b).

In the present work, the biological responses to the eutectoid ceramic were studied using ahMSCs, which have been frequently used to elucidate the responses of bone cells to biomaterials [29,30].

The obtained eutectoid had a bioactive surface that displayed the formation of a calcium-phosphate layer after exposure to a human bone marrow culture (Figure 3). This apatite-like layer could play an essential role in the primary chemical bonding of materials for implantations into receptor osseous tissues.

The results obtained for the proliferation and growth of ahMSCs on the eutectoid ceramic proved that after the experiments, cells had homogeneously distributed over the specimens and were well-attached and had uniformly colonised the sample surface. This indicates that the material developed herein is biocompatible, and offers high colonisation and proliferation rates.

The MTT assay demonstrated that the eutectoid ceramic had a limited deleterious effect on cell viability after the first 24 h in culture, possibly because cells had to adapt to the new environment whose ion concentration differed considerably from the normal culture medium. Moreover, cells had to adhere to a new substrate with roughness and stiffness properties, which was different from the culture flask plastic used in previous cultivation steps.

In the proliferation study, the culture control progressed linearly with time, but the proliferation rate was quite modest (the cell number did not double in 1 week). The new eutectoid biomaterial consists of 58.43 wt % of CaO, so a high level of calcium ion is released into the environment until the 12th day (Figure 8). High levels of Ca concentrations (up to 300 mg/L) have proved cytotoxical, but low (2–4 mM) and medium (6–8 mM) Ca concentrations are suitable for proliferation [31]. Low levels of proliferation and spherical cell shape of the first week can be explained due to the increasing release of Ca at the beginning of the experiment. The addition of OM increased this rate by about ~15%–35%, possibly because OM was supplemented with β-glycerophosphate, which is an important source of inorganic phosphate (Pi). In fact, Pi is known as an important signalling molecule for not only cell differentiation, but also for cell proliferation, by regulating the proteins involved in the control of cell cycle and DNA synthesis [32,33].

The MTT results (Figure 4) showed that the biomaterial influenced cell proliferation by stimulating it. In fact, from week 2, the proliferation rate of the cells seeded on the eutectoid ceramic overtook that of the control and remained constant throughout the incubation period. Moreover, this effect increased with the addition of OM, possibly due to the concentration of calcium and phosphate, an ion that the eutectoid ceramic released in the culture medium. They can both stimulate cell proliferation, and this effect was enhanced by the presence of osteogenic medium when an extra Pi source was included. The eutectoid ceramic probably also had a particular surface that favoured cell adhesion, as well as a particular chemical structure that resulted in a combined set of ions released in the culture medium (Figure 8) that induced cell proliferation.

Several studies [34,35] have shown that surface roughness is an important parameter in basic cell biological responses as it improves cell attachment and proliferation. ahMSCs had significantly higher levels of cell attachment on rough sandblasted surfaces and more irregular morphologies than on smooth surfaces. The eutectoid ceramic samples studied herein exhibited a rough surface morphology (see Figure 1b). Thus the material’s surface properties can help to promote osteoblast-like cell attachment.

Cell culture does not allow the analysis of possible differences in the response that results from the two distinct phases present in the sample (Nurse’s A-phase and silicocarnotite). Under osteogenic conditions, the levels of viable cell adhesion on the crack-free specimen surface were comparable with other apatite surfaces in the osteogenic medium [36,37].

Adhesion and spread of cells on the material surface not only manifest interactions between cells and materials, but also regulate cellular functions, such as proliferation, migration and extracellular matrix production [38,39]. In view of these reasons, ahMSCs were seen to attach to the eutectoid ceramic disc by SEM.

The SEM images of the eutectoid ceramic soaked in GM showed how the ceramic surfaces were covered by small nodules as early as seven post-incubation days, and the SEM-EDX microanalysis confirmed the nodules’ nature to be calcium-phosphate precipitates. With this method, we were able to observe on day 7 that cells continued to adhere to the eutectoid ceramic material surface. Although some cells still looked round and globular, others were polygonal in shape. The cell number grew and an increase in cytoplasmatic prolongations (filopodia) and cell-to-cell interactions was recorded. During this period, presence of mineralisation nodules was also considerable and suggested the continuity of their deposition on the eutectoid ceramic surface. Moreover, granules were seen above the cells that came into contact with their membrane, so granules formed at the same time as cells proliferated and covered the material surface.

Later on day 21, a general increase in the number of cells and interconnections was observed. There were more interconnections between cells; cells began to adopt a fibroblastic-like shape (fusiform) and they covered almost all the material pieces. Intercellular spaces were filled with a fibrillar framework (with no organisation), which corresponded to the extracellular matrix produced by cells. OM also stimulated cell proliferation and not only differentiations as the surface of the pieces in this case were better covered by cells. The SEM data confirmed the data recorded during the MTT, when proliferation was enhanced by addition of OM. Finally on day 28, the eutectoid ceramic pieces were totally covered by cells, and there were no differences between the GM and OM samples. At all the time points, cells adhered to the biomaterials directly and firmly, and also to the neo-formed apatite layer (granules).

At this point, it is important to note that the inductive effect of the eutectoid ceramic on ahMSCs adhesion and proliferation was favoured by the release of Si and Ca ions into the medium (Figure 8), and by the formation of the aforementioned Ca-P layer (Figure 3). The processes of osteoblastic proliferation and differentiation are not only time-dependent, but also regulated by various switches including extracellular matrix (ECM) maturation and hormonal or mechanical stimuli [40]. Extracellular Ca plays an important role in the regulation of osteoblastic proliferation and differentiation by changing the expression of specific Ca channel isoforms on osteoblasts, and has been accepted as a coupling factor between osteoclasts and osteoblasts [41]. In addition, the Ca/P ratio obtained after 14 days in GM medium for the eutectoid material is superior to the ion release of standard materials such as HA and TCP, as well as silicon doped materials as shown in Table 1. This is mainly because the Ca/P ratio of the raw AS material is much higher than those standard ratios, but never reaches the cytotoxic levels after the in vitro assay [31].

Mineral crystal deposition was also studied by Alizarin Red staining. During the 14- and 28-day incubations with the biomaterials and the GM medium, only a few small spots spread over the monolayer. Strong mineralisation occurred during the 28-day incubation, when cells were incubated with the eutectoid ceramic and the OM medium, and displayed significant differences compared to the control samples (Figure 6). Mineralisation took place in the samples incubated with the eutectoid ceramic due to the deposition of an enriched extracellular matrix in the mineral-nucleating proteins, which was able to organise and direct calcium phosphate along the collagen fibre precipitates, most of which derived from the biomaterial dissolution products and osteogenic supplements. Quantification was roughly confirmed by the qualitative analysis. ALP activity in the samples incubated with the AS ceramic on day 28 was greater than enzyme activity in the control cells. Recent studies indicate that silicon promotes cell proliferation, ALP expression and mineralization [43,44]. Mineralization begins with hydroxyapatite formation in the matrix vesicles budding from osteoblasts. Hydroxyapatite is formed from Ca incorporated by the annexin calcium channel and from inorganic phosphate Pi [45]. In this chase, the Ca releasing rate is obviously high from AS ceramic. Moreover, the biomaterial is constantly providing phosphorus ions to the medium since day 4 (Figure 8), benefiting the formation of mineralized nodules before alkaline phosphatase is active.

The cell surface markers analysis showed that the cells which had seeded on the eutectoid ceramic present had a reduced expression of CD 105, a marker expressed in ahMSCs whose drop in expression is considered a sign of differentiation programme activation (Figure 7). We highlight that the CD 90 marker expression was reduced for the material and control samples by day 21 in the presence of OM. There could be several factors behind the expression of the osteoblastic markers noted in the control samples. First of all, the chosen population could be constituted by many cells already compromised or contaminated by other cell types. In fact, samples of marrow extruded from bone can include osteoblast precursors eluted from either trabecular bone or the inner surface of the bone itself. Even culture conditions can influence the spontaneous differentiation of ahMSCs. DMEM contains Phenol Red as a pH indicator, which is able to mimic estrogens, potent inducers of osteoblast differentiation. Moreover, the content of Fetal bovine serum (FBS) growth factors preferentially induces ahMSC differentiation towards the osteogenic lineage instead of towards the chondrogenic or adipogenic lineage. These observations highlight the need to better characterise the cells used and to improve the culture conditions in order to verify normal differentiation programme induction when cells remain in culture for a long period and reach confluence.

## 4. Materials and Methods 

### 4.1. Biphasic Ceramic Preparation and Characterisation

Samples were prepared from Nurse’s A and silicocarnotite powders, which were used as starting materials. Details of the technique and characterisation of the starting materials can be found in previous publications [21,22,23,24].

A mixture of 28.39 wt % Nurse’s A-71.61 wt % silicocarnotite was prepared. Firstly, Nurse’s A and silicocarnotite powders were ground to an average particle size of ~20 µm. The desired proportions of each component were weighed on an analytical balance and thoroughly mixed with PSZ (partially stabilized zirconia)-zirconia balls. After drying, samples were isostatically pressed in bars at 200 MPa. The pellets obtained from the bars were placed into small platinum foil crucibles, which were suspended from a platinum wire in the hot zone of an electrical furnace with an electronic temperature controller (±1 °C). Pellets were heated to 1550 °C for a total time period of 144 h (6 days), with quenching in liquid-nitrogen, milling, pressing and reheating every 24 h. The ceramic was heated to 1500 °C/24 h and slow-cooled to room temperature at a rate of 6 °C/min. This combined heat treatment procedure was required to ensure that equilibrium conditions were achieved. The heat treatment temperatures were carefully selected by considering the information provided by the Nurse’s A-phase-silcocarnotite [25] subsystem that existed in the binary system of Ca_3_(PO_4_)_2_-Ca_2_SiO_4_.

The material was characterised by X-ray diffraction patterns (XRD, AXS D8 Advance, Bruker, Karksruhe, Germany) and compared with the database provided by the Joint Committee on Powder Diffraction Standards (JCPDS). The ceramic’s microstructure was studied by Scanning Electron Microscopy (SEM, S-3500N, Hitachi, Ibaraki, Japan) in an Energy Dispersive X-ray Spectrometer. The final samples were cut from the obtained bars, which had a diameter of 7 mm and a thickness of 3 mm.

### 4.2. Bone Marrow-Derived ahMSCs Isolation, Culture and Subculture

Undifferentiated multipotent ahMSCs were isolated from the heparinised bone marrow collected from three healthy male human volunteers (50 mL/patient), who underwent elective surgical procedures by percutaneous direct aspiration from the iliac crest. All the procedures were approved by the Institutional Ethical and Clinical Trials Committee (V. Arrixaca University Hospital of Murcia). Informed consent was obtained from all the volunteers. Details of the method and technique used to obtain, subculture and characterise cells prior to seeding on the biomaterials can be found in previous publications [46]. Cells from the third pass (P3) were used in all assays.

### 4.3. Phase Contrast Optical Microscopic Observations

To control the evolution of ahMSCs cultures, the cells isolated and attached to plastic were observed under an inverted phase-contrast light microscope (Nikkon Elipse TS, Tokyo, Japan), and their morphological changes, adhesion properties on plastic (control) and growth under the indirect effect of the tested materials were recorded. For this purpose, cells were seeded at a density of 1.0 × 10^4^ cells/well in 24-well culture plates, and were cultured in GM and OM under the same controlled culture conditions (37 °C, 95% humidified atmosphere and 5% CO_2_). Then, the sterile biomaterial samples were placed in the upper chambers of 8-µm pore-size cell culture inserts (BD Bioscience, San José, CA, USA), and were incubated in wells in the presence of ahMSCs for 48 h, and for 7, 15 and 21 days. GM was renewed three times a week. Another seeded culture plate with no material was used as the control.

### 4.4. Seeding ahMSCs on Biphasic Material

To study the biocompatibility and behaviour of the isolated ahMSCs on Nurse’s A-phase-silicocarnotite ceramics, two kinds of cell culture media were used: (i) basal GM, used for cell isolation and expansion, which consists in αMEM supplemented with 10% of heat-inactivated FBS and penicillin/streptomycin (100 U·mL^−1^ and 100 µg·mL^–1^, respectively); (ii) osteogenic-inducing medium (OM), which consists in GM with an osteogenic supplement (OS) composed of L-ascorbic acid-2-phosphate (0.2 mM; Sigma, St. Louis, MO, USA) dexamethasone (10 nM; Sigma) and β-glycerolphosphate (10 mM; Merck, Darmstadt, Germany).

**Figure 9:**
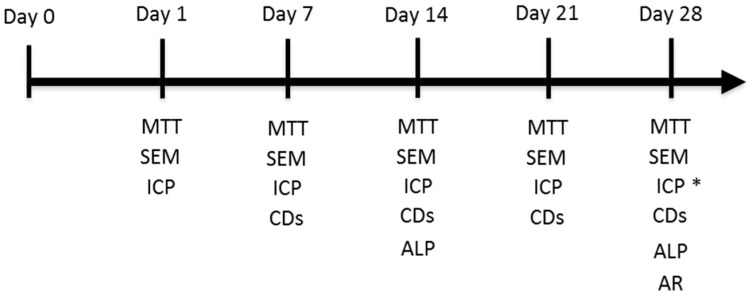
Experimental set-up (* only in GM).

Initially, ceramic discs were cleaned with pressured air, rinsed several times with PBS (pH 7.4), dried at 37 °C and sterilised at low temperature by gas plasma (Sterrad-100S™, ASP Irvine, Irvine, CA, USA). Discs were pre-wetted in GM for 2 h prior to seeding, and were placed inside the wells of a 96-well culture plate (one disc per well). Then 5.0 × 10^3^ cell·cm^–2^ were gently and statically seeded by pipetting a droplet of 10 μL of the cell suspension on top of each sterilised disc and incubated at 37 °C for 1 h to allow cells to attach to the material’s surface. New GM was then carefully added (until 0.2 mL) for further incubations. The control cultures included the cells grown on plastic in the absence of the material. Incubation was carried out under the aforementioned standard conditions (37 °C, 5% CO_2_ and 95% humidity). Discs were exposed to GM for 24 h and for 7, 14, 21 and 28 days. Starting on day 21, when cells were calculated to reach confluence, half the samples were changed from GM to OM, and cell behaviour was analysed on days 21 and 28 by a proliferation assay, SEM and osteoblastic phenotype expression, according to the following set-up (Figure 9). All the quantitative assays were carried out in triplicate.

### 4.5. Morphological Evaluation: Scanning Electron Microscopy

In order to assess cell-material interactions and their continuing effect on the behaviour of ahMSCs, in both cell adherence and growth terms, and from an ultrastructural viewpoint, study periods that lasted 24 h, and 7, 14, 21 and 28 days in GM, and 21 and 28 days in OM, were established for the SEM examinations. Before the cell culture studies, and in order to recognise the seeded ceramic surface, one of the sample faces was carefully impressed with an electrical marker. Specimens were prepared according to previously reported SEM protocols [2,39].

### 4.6. Proliferation Assay

The increment in cell number on the material’s surfaces using both media (GM and OM) was quantified spectrophotometrically by a tetrazolium salt MTT assay. Details on the method and technique can be found in previous publications [46].

### 4.7. Osteogenic Differentiation Assay

The effect of eutectoid discs on the early osteogenic differentiation of ahMSCs was assessed by ALP specific activity. Next, 5.0 × 10^3^ cell·cm^–2^ were cultured on the discs using GM for 14 and 28 days or OM for 28 days. At the end of this time, cell discs were washed twice with PBS. Alkaline phosphatase (ALP) activity was determined at 405 nm using p-nitrophenylphosphate (pNPP) (Sigma) as the substrate, and total protein contents were measured by the bicinchoninic acid (BCA) protein assay (Pierce, Rockford, IL, USA) using bovine serum albumin (BSA) (Pierce Bovine Serum Albumin) as the standard.

### 4.8. Calcium Deposition (Mineralisation)

The ability of cells to produce a mineralised matrix and nodules is important for the development of materials for bone regeneration.

Presence of calcium deposition or nodules of mineralisation in cultures was evaluated qualitatively and quantitatively by the selective binding of Alizarin Red S (Osteogenesis kit assay, Millipore, Bredford, MA, USA) to calcium salts on day 28 in both cell media and following the manufacturer’s protocol. Briefly, AS ceramic scaffolds were placed in transwell inserts in 24-well plates and ahMSCs were seeded at the bottom of the wells at a density of 5 × 10^3^ cells cm^–2^ and incubated at 37 °C in a humidified atmosphere consisting of 95% air and 7.5% CO_2_.

After culturing, scaffolds and inserts were removed and the cells were rinsed three times in phosphate-buffered saline (DPBS), fixed with 8% paraformaldehyde at RT for 15 min, and stained with Alizarin Red Stain solution for 20 min. Stained areas were visualised under an optical microscope and images were acquired. The differentiated cells that contained mineral deposits were stained bright red by Alizarin Red solution.

The quantitative Alizarin Red staining analysis was carried out by determining the absorbance at 405 nm of a set of known Alizarin Red concentrations and comparing these values to those obtained from unknown samples. Briefly, 400 µL of 10% acetic acid were added, after acquiring images, to each 24-well plate well and samples were incubated for 30 min with shaking. The monolayer was gently detached and transferred with acetic acid solution to a 1.5 mL microcentrifuge tube and vigorously mixed (Vortex) for 30 s. Samples were sealed with parafilm to avoid evaporation and were heated to 85 °C for 10 min by placing them in a heat bath. Tubes were then transferred to ice for 5 min. Centrifugation at 20,000× *g* for 15 min allowed calcium deposit recovery. While centrifuging, Alizarin Red standards were prepared following the manufacturer’s protocol. After one centrifugation step, 400 µL of the supernatant of each sample was withdrawn and transferred to a new 1.5 mL microcentrifuge tube. pH was neutralised with 150 µL of 10% ammonium hydroxide. Samples were transferred to a transparent-bottomed 96-well plate and absorbance was red at 405 nm in a plate reader (Multiscan MCC 340, Lab Systems, Vantaa, Finland). The set of Alizarin Red known concentrations was plotted against their values of absorbance to obtain a standard curve. To determine the concentration of the unknown sample, its absorbance value was plotted to the standard curve.

### 4.9. Monitoring Surface Markers in the Cells Seeded on the Biphasic Ceramic

A flow cytometry analysis was performed to analyse the expression of representative surface markers CD105, CD90 and CD73 for the ahMSCs seeded on the eutectoid ceramic to be compared with the control after 7, 14, 21 and 28 days of growth in basal GM, and after 21 and 28 days in OM.

Next, 5000 cells·cm^–2^ were seeded on the biomaterials. At the end of every week, cells were trypsinised and processed, then transferred to a 50 mL tube and centrifuged at 1000 rpm for 10 min at 4 °C, and kept on ice during manipulation. Finally, cells were resuspended in a volume of 1% FBS in DPBS to obtain a concentration of 2800 cell·µL^–1^.

Then, 45 µL of the cell suspension was aliquoted into as many tubes as required. Antibodies were diluted at the recommended (and pre-standardised by our laboratory) dilution in DPBS. Blank: 45 µL of cell suspension and 5 µL of DPBS. Sample: 45 µL of cell suspension and 5 µL of each antibody: 5 µL of CD90-APC (1/100), 5 µL of CD105-FITC (1/10) and 5 µL of CD 73-PE (1/10) (typical MSC markers). Negative control: 45 µL of cell suspension and 5 µL of each antibody: 5 µL of CD45-FITC (1/10) and 5 µL of CD34-PE (1/10) (typical haematopoietic markers). Isotype control: 45 µL of cell suspension and 5 µL of each isotype control: 5 µL of APC-Mouse IgG1k (1/10), 5 µL of PE-Mouse IgG1k (1/10) and 5 µL of FITC-Mouse IgG2ak (1/10). After passing the cleaning solution (Coulter Clenz™, Fisher, IL, USA) in the cytometer, the samples that contained the cells marked with antibodies and blanks were passed in sequence. Data were retrieved with a Beckman Coulter Navios flow cytometer and analysed with the Navios flow cytometry software. All the antibodies were purchased from Becton, Dickinson Co., Franklin Lakes, NJ, USA.

### 4.10. The Ions Released from the Eutectoid Ceramic

In order to measure the silicon (Si), calcium (Ca) and phosphorous (P) released from the eutectoid ceramics, four discs were added in 1 mL of GM at 37 °C under static conditions to mimic the cell culture conditions. Then, the medium was collected on days 0.5, 1, 4, 7, 14, 21, 28 and 36. The Si, Ca and P concentrations in GM were measured by Inductively Plasma Optical Emission Spectroscopy (ICP-OES, PerkinElmer Optima 2000, Waltham, MA, USA). An analysis of the complete GM was taken as a reference. The instrument’s limits of detection (LoD) for the elements of interest were 0.05 for Si, 0.10 for Ca and 0.20 for P/ppm.

### 4.11. Statistical Analysis

A repeated measures analysis of variance (ANOVA) was run at a level of statistical significance of *p* < 0.05. This was possible because, after applying natural logarithm transformation to the data, they fulfilled the conditions of homoscedasticity and sphericity required for the analysis.

## 5. Conclusions

By means of the subsystem Nurse’s A-phase-silicocarnotite, we designed and processed a material with a chemical composition of the invariant eutectoid point and lamellae morphology.

The results obtained for the proliferation, growth and osteoblastic differentiation of adult human mesenchymal stem cells on the material proved that the biphasic ceramic developed herein is not cytotoxic, cells strongly adhere to the substrate and quickly proliferate on it, and it is capable of initiating the osteogenic differentiation process with both GM and OM.

The essential role of a co-treatment with silicon and calcium as important elements which enhance ahMSCs activity and phosphorous-based biomaterials was highlighted. Additionally, surface roughness is an important parameter in the basic biological responses as it improves cell attachment, proliferation and differentiation of undifferentiated cells.

The biphasic Nurse’s A-phase-silicocarnotite ceramic that we produced could serve as a promising platform for hard tissue regeneration through tissue engineering, although in vivo animal implantation could help to determine the final clinical applications.

## Figures and Tables

**Table 1:** Ca/P ratio in the AS material as well as some calcium phosphate (CaP) and Si doped CaP materials.

**Ca/P Ratios**	**Raw Materials**				
	AS	TCP	HA	Si-TCP [2,42]	Si-HA [43]
Ca/P ratio	6.0	1.5	1.67	1.51/1.53	1.69
	**Release after 14 Days in GM Medium**				
	AS	TCP [2,42]	HA [43]	Si-TCP [2,42]	Si-HA [43]
Ca/P ratio	4.22	1.09	0.32	0.93/0.87	0.47
